# Substance use among individuals with mild intellectual disability or borderline intellectual functioning in residential care: Examining the relationship between drinking motives and substance use

**DOI:** 10.1111/jar.12578

**Published:** 2019-03-07

**Authors:** Esmée P. Schijven, Robert Didden, Roy Otten, Evelien A. P. Poelen

**Affiliations:** ^1^ Research & Development Pluryn Nijmegen The Netherlands; ^2^ Behavioural Science Institute Radboud University Nijmegen The Netherlands; ^3^ Trajectum Zwolle The Netherlands; ^4^ REACH Institute Arizona State University Phoenix Arizona

**Keywords:** alcohol use, borderline intellectual functioning, drug use, mild intellectual disabilities, motives for substance use

## Abstract

**Background:**

This study examined the relationship between substance use motives (i.e., social, conformity, coping and enhancement) and substance use in individuals with mild intellectual disability or borderline intellectual functioning (MID‐BIF).

**Method:**

Data were collected among 163 clients with MID‐BIF using interactive questionnaires with visual cues on a tablet with a web application.

**Results:**

Results show that social motives were positively related to frequency of alcohol use, while conformity, coping and enhancement motives were positively related to severity of alcohol use. Results for drug use show that social motives were positively related to frequency of cannabis and hard drug use and that conformity motives were negatively related to frequency of cannabis use. Coping motives were positively related to severity of drug use.

**Conclusions:**

Insight in substance use motives should be used when adapting interventions, as it could contribute to the prevention and reduction of substance use disorders in individuals with MID‐BIF.

## 1 INTRODUCTION

Individuals with mild intellectual disabilities or borderline intellectual functioning (MID‐BIF; IQ between 50 and 85 and limitations in social adaptive skills; American Psychiatric Association, 2013) are using alcohol and drugs similar to their peers without MID‐BIF (Poelen, Schijven, & Vermaes, 2015; van Duijvenbode et al., 2015). Substance use—especially the use of alcohol—is relatively common in this group and often coincides with psychological, social and/or financial problems (Didden, 2017). Compared to people without MID‐BIF, people with MID‐BIF have a higher risk for developing substance use disorders (Burgard, Donohue, Azrin, & Teichner, 2000; McGillicuddy, 2006; van Duijvenbode et al., 2015). Substance use disorders are determined based on criteria pertaining the amount of use, interpersonal or social problems, continued use despite risky consequences, and the advance of tolerance and withdrawal symptoms (APA, 2013). Particularly among people with MID‐BIF in combination with behavioural problems, delinquent behaviour or psychiatric comorbidity prevalence rates of substance use disorders are high (see e.g., Chaplin, Partsenidis, Samuriwo, Underwood, & McCarthy, 2014; van Duijvenbode et al., 2015).

Next to being adapted to the target group, treatment of substance use disorders in individuals with MID‐BIF should be based on risk factors of substance use as well as on motives for substance use. According to the “Motivational model of alcohol use” (Cox & Klinger, 1988), alcohol use serves different functions and is driven by different needs. Motives for alcohol use are considered crucial for the final decision whether to drink or not (Cooper, 1994; Cox & Klinger, 1988; Kuntsche & Kuntsche, 2009), and therefore, it is essential to understand motives for substance use. According to Cox and Klinger (1988), the decision whether to drink or not is based on rational and emotional processes, since it depends on what (affective) changes someone expects. Obviously, direct and indirect effects of substance use play an important role in this expectation. Use of alcohol, for example, has a direct effect on the emotional state of individuals. However, alcohol can also have an indirect effect, for example, if drinking alcohol improves contact with peers through changes in someone's social inhibition (Kuntsche & Cooper, 2010).

The “Motivational model of alcohol use” (Cox & Klinger, 1988) posits that individuals drink to obtain positive outcomes or to avoid negative consequences. In addition, they are motivated to drink alcohol because of internal rewards, such as improvement of the state of mind, or because of external rewards, such as social approval or acceptance by others (Kuntsche, Knibbe, Gmel, & Engels, 2005; Kuntsche & Kuntsche, 2009). Four different motives for alcohol use have been distinguished (Cooper, 1994; Kuntsche & Kuntsche, 2009). Individuals with *social motives* use alcohol at social events in order to confirm social relations or to enjoy social occasions (i.e., external, positive reinforcement) (Kuntsche & Kuntsche, 2009; Kuntsche, Le Mével, & Berson, 2016). Individuals with *conformity motives* use substances to prevent (social) rejection and to be part of a group (i.e., external, negative reinforcement) (Kuntsche & Kuntsche, 2009; Kuntsche et al., 2016). Individuals with *coping motives* use substances to regulate their negative emotions and to deal with (emotional) problems (i.e., internal, negative reinforcement) (Kuntsche & Kuntsche, 2009; Kuntsche et al., 2016). Finally, individuals with *enhancement motives* use substances to create a positive mood or to have fun (i.e., internal, positive reinforcement) (Kuntsche & Kuntsche, 2009; Kuntsche et al., 2016).

Studies on the relationship between the four motives and alcohol use show that social motives are related to irregular and non‐problematic use of alcohol (see e.g., Cooper, 1994; Mezquita, Stewart, & Ruipérez, 2010). The relationship between conformity motives and alcohol use is more ambiguous and seems to depend on the outcome measure, environmental factors, phase of alcohol use and age. For instance, some studies found a negative relationship between conformity motives and quantity of alcohol consumption, while others found a positive relationship between conformity motives and alcohol‐related problems (Kuntsche, Knibbe, Engels, & Gmel, 2007; Kuntsche et al., 2005). In adolescents, conformity motives may be beneficial in a positive social context to connect with a group of friends, but may instigate a negative effect in a social context with friends who display risky behaviour. In contrast, coping motives and conformity motives are associated with a risk for problematic alcohol use, such as heavy alcohol use and alcohol‐related problems (Cooper, 1994; Mezquita et al., 2010; van der Zwaluw, Kuntsche, & Engels, 2011).

The four motives have not yet been studied in people with MID‐BIF. In addition, only the motives of alcohol use were examined and motives for drug use have not been included in studies among individuals without MID‐BIF. Since these people have a higher risk for problematic substance use and motives can be an important factor for prevention and treatment of substance use disorders, it is of importance to investigate the relationship between the motives and substance use in this target group.

## 2 METHODS

### 2.1 Participants, setting and procedure

This study was conducted in eight residential settings located in different areas in the Netherlands that provide intramural care, education/work and recreation to people with MID‐BIF and behavioural problems. Participants were 163 individuals with MID‐BIF who had used alcohol and/or drugs in the past. This was assessed using self‐report. Data on 163 participants had been collected in two earlier studies. In the first study (see Poelen, Schijven, Otten, & Didden, 2017), 204 clients were approached spread over three care facilities. Fifty‐eight individuals did not return the informed consent forms by their parents or legal representatives, 23 individuals were not included because they were not reaching inclusion criteria (like psychological unstable, did not want willing to participate), and 17 participants were excluded from analyses as they had never used alcohol and/or drugs. Hence, from this study 106 (64%) participants were included for analyses in this study. The remaining 57 (35%) individuals participated in a study that evaluated the effectiveness of an intervention for problematic substance use (see Schijven, Engels, Kleinjan, & Poelen, 2015). All these participants had used alcohol and/or drugs before. Data were collected with interactive questionnaires with visual cues on a tablet with a web application. Questions were read out loud by trained researchers (i.e., university master students) and were clarified with a simple explanation or examples if necessary. In addition, case files were used to collect information about IQ and psychiatric diagnoses (DSM‐IV‐TR, DSM‐5). Participants and, in prevailing cases, their parent(s) or legal representative had given written consent to participate in this research. They were informed both orally and in writing about the purpose of the study in which anonymity was assured. People with MID‐BIF who were psychologically unstable, for example, because of a psychotic episode, were excluded. The study was approved by the Ethics Committee of the Faculty of Social Sciences of the Radboud University at Nijmegen (number: ECSW2015‐0903‐303).

The mean age of the participants was 18.9 years (range: 11–30; *SD* = 4.4), 73% were male (*n* = 119) and 27% were female (*n* = 44). The mean total IQ (measured with the WAIS or WISC) of the respondents was 71.6 (range: 52–85; *SD* = 7.8). Of the participants of whom information about total IQ scores was available, 37% (*n* = 47) had a mild intellectual disability (MID; IQ 50–70) and 63% (*n* = 60) had borderline intellectual functioning (BIF; IQ 70–85). Out of 163 participants, 55% had one or more DSM diagnose(s) of which pervasive developmental disorder (PDD; 23%), attention deficit hyperactivity disorder (ADHD; 23%), attachment disorders (14%) and oppositional‐defiant disorder (ODD), and conduct disorder (CD) (14%) were the most common. In 22% (*n* = 36) of participants, their case files did not contain total IQ scores and information about psychiatric diagnoses, but all participants received care from a setting that is specifically targeted at care for people with MID‐BIF.

### 2.2 Instruments

Substance use was measured with the Substance Use and Misuse in Intellectual Disability‐Questionnaire (SumID‐Q; VanDerNagel, Kiewik, Dijk, Jong, & Didden, 2011). This instrument provides opportunities for an open and friendly way to discuss the use of substances with people with MID‐BIF. The SumID‐Q is modular, and participants only need to answer follow‐up questions about the substances that they are familiar with (VanDerNagel et al., 2011). In addition, the SumID‐Q was adapted to the target group by simple phrasing and use of language among others. Current use of alcohol, cannabis and/or hard drug (in the Netherlands, the opium law distinguishes hard drugs from soft drugs. Hard drugs are defined as drugs with significant, unacceptable high risks for health while soft drugs are defined as drugs with a reduced risk. Examples of hard drugs are cocaine, ecstasy and heroine) of the participants was measured with the item: “Have you used … during the past month?” (1) “yes” or (2) “no.” Frequency of alcohol, cannabis and/or hard drug use was measured with the item: “How often do you use?” (1) “never” to (6) “almost every day.” Problematic substance use was measured with the Alcohol Use Disorder Identification Test (AUDIT; Babor, Higgins‐Biddle, Saunders, & Monteiro, 2001) and the Drug Use Disorders Identification Test (DUDIT; Berman, Bergman, Palmstierna, & Schlyter, 2003). The two questionnaires each consist of ten items concerning the frequency and amount of use, symptoms of dependency and problems related to the use of substances. An example item is “How often were you in need of alcohol/drugs in the morning after having a severe amount of alcohol/drugs on the previous night?” The items were answered on a five‐point scale ranging from (1) “never” to (5) “almost every day.” The total scale score was calculated by averaging the item scores. In addition, participants with a score of 8 or higher on the AUDIT were categorized as “problematic drinker” and participants with a score of 5 or higher on the DUDIT were categorized as “problematic user.” These cut‐off points are determined on the basis of research in people without MID‐BIF (Babor et al., 2001; Berman et al., 2003). The items of the AUDIT and the DUDIT have been shown to be applicable in people with MID‐BIF (van Duijvenbode, Didden, Korzilius, & Engels, 2016). In the present study, the Cronbach's alpha for the AUDIT and the DUDIT was 0.70 and 0.86, respectively.

Motives for alcohol use were measured with the Drinking Motive Questionnaire Revised Short Form (DMQ‐R‐SF; Kuntsche & Kuntsche, 2009). This questionnaire consists of 12 items and measures the four motives for alcohol use (i.e., “social,” “conformity,” “coping” and “enhancement” motives), by means of the question: “Why do you drink alcohol?”. Example items are Because it makes me feel happy and because it helps me when I feel bad. Items were answered on a three‐point scale (1) never, (2) sometimes and (3) almost always. The motives for drug use were determined using the DMQ‐R‐SF, with similar questions but then referring to the use of drugs (i.e., “Why do you use drugs?”). The items relating to drug use were completed by participants who used hard drugs and not by the respondents that only use cannabis. Cronbach's alpha's for motives for alcohol use were between 0.64 and 0.86; for drug use, they were between 0.77 and 0.89.

### 2.3 Statistical analyses

First, we performed descriptive analyses of substance use (current use, frequency of use and AUDIT/DUDIT scores) and motives for substance use (social, conformity, coping and enhancement). Before we tested the relationship between substance use motives and alcohol and drug use, we examined the measurement model of the motive items using confirmatory factor analyses in Mplus 7.2 (Muthén & Muthén, 1998–2012). The maximum likelihood (ML) estimator was used to estimate parameters in the model. The chi‐square and the *p*‐value, the Comparative Fit Index (CFI; Bentler, 1990) and the root mean square error of approximation (RMSEA; Steiger, 1990) were used to assess the goodness of fit of the model. RMSEA values of below 0.08 and CFI values of above 0.90 reflect an acceptable fit of the model to the data (Hu & Bentler, 1999). We examined differences in substance use between participants with mild intellectual disabilities (MID; IQ 50–70) and participants with borderline intellectual functioning (BIF; IQ 70–85), using chi‐square tests and *t* tests. Pearson correlations were calculated to explore relationships between motives and substance use. In addition, multivariate linear regression analyses were conducted to examine multivariate relationships between motives and substance use. Multivariate linear regression analyses were conducted in SPSS 21 to examine multivariate relationships between motives and substance use.

## 3 RESULTS

### 3.1 Descriptive statistics substance use

Of the participants, 62% currently used alcohol, 34% used cannabis and 20% used hard drugs (see Table 1). Of all participants, 23% (*n* = 38) used both alcohol and drugs in the last month. In total, 41% of the participants scored above the cut‐off criterion for problematic use (score of 8 or higher) on the AUDIT, and 45% (*n = *73) scored on the cut‐off criterion for problematic use (score of 5 or higher) on the DUDIT. For both alcohol and drug use, 25% (*n* = 40) had a score above the cut‐off point.

**Table 1:** Descriptive statistics of substance use among people with mild intellectual disability or borderline intellectual functioning

	MID (*n* = 47)	BIF (*n* = 80)	Total (*n* = 163)	Difference
Current use	%	%	%	
Alcohol	63.8 (*n* = 30)	62.5 (*n* = 50)	62.0 (*n* = 101)	*χ* ^2^ (1) = 0.02, *p* = 0.881
Cannabis	34.0 (*n* = 16)	32.5 (*n* = 26)	33.7 (*n* = 55)	*χ* ^2^ (1) = 0.03, *p* = 0.858
Hard drug	21.3 (*n* = 10)	18.8 (*n* = 15)	20.2 (*n* = 33)	*χ* ^2^ (1) = 0.12, *p* = 0.730
Frequency of use	Mean (*SD*)	Mean (*SD*)	Mean (*SD*)	
Alcohol	2.7 (1.0)	2.7 (1.0)	2.7 (1.0)	*t*(125) = 0.53, *p* = 0.599
Cannabis	1.7 (1.7)	2.0 (1.6)	2.0 (1.6)	*t*(125) = −0.85, *p* = 0.395
Hard drug	1.1 (1.5)	1.2 (1.3)	1.2 (1.4)	*t*(125) = −0.42, *p* = 0.672
Severity of use	Mean (*SD*)	Mean (*SD*)	Mean (*SD*)	
AUDIT	8.5 (8.3)	7.3 (6.3)	7.4 (6.8)	*t*(125) = 0.94, *p* = 0.348
DUDIT	6.7 (9.5)	6.6 (7.8)	6.9 (8.7)	*t*(125) = 0.04, *p* = 0.965
Motives for alcohol use				
Social	1.9 (0.6)	2.0 (0.5)	2.0 (0.5)	*t*(125) = −1.38, *p* = 0.170
Conformity	1.3 (0.5)	1.2 (0.4)	1.2 (0.4)	*t*(125) = 1.04, *p* = 0.298
Coping	1.6 (0.7)	1.4 (0.6)	1.5 (0.6)	*t*(125) = 1.70, *p* = 0.091
Enhancement	1.8 (0.7)	1.6 (0.5)	1.6 (0.5)	*t*(125) = 1.34, *p* = 0.184
Motives for drug use				
Social	1.8 (0.7)	1.7 (0.6)	1.6 (0.6)	*t*(68) = 0.79, *p* = 0.431
Conformity	1.4 (0.7)	1.3 (0.5)	1.3 (0.5)	*t*(68) = 0.74, *p* = 0.460
Coping	2.0 (0.9)	1.8 (0.7)	1.8 (0.8)	*t*(68) = 0.96, *p* = 0.337
Enhancement	2.0 (0.7)	1.9 (0.7)	1.9 (0.7)	*t*(68) = 0.57, *p* = 0.572

Note

BIF: borderline intellectual functioning; MID: mild intellectual disability.

No significant differences were found in motives, current use, frequency of use and severity of use between participants with MID and participants with BIF (also see Table 1). There were no significant differences in age (*t*(125) = 1.94, *p* = 0.054) and sex (*χ* ^2^(1) = 0.08, *p* = 0.782) between participants with MID and participants with BIF. The only significant difference between males and females was found for coping motives (*t*(161) = −2.26, *p* = 0.025) and enhancement motives (*t*(161) = −2.19, *p* = 0.030) on alcohol use. Females scored higher on both these motives than males. Other than that, there were no significant differences in motives, current use, frequency of use and severity of use between males and females (*t* test ranged between *t*(161) = −2.22, *p* = 0.824 and *t*(161) = 0.91, *p* = 0.362).

### 3.2 The relationship between motives and substance use

We examined the measurement model of the four‐factor structure of the motives items before testing the relationship between motives and substance use (Table 2). Both models showed a good fit to the data (*χ* ^2^(48, *N* = 163) = 79.52, *p *< 0.01; RMSEA = 0.063; CFI = 0.958 for drinking motives and *χ* ^2^(48, *N* = 90) = 65.88, *p *< 0.05; RMSEA = 0.064; CFI = 0.975 for drug use motives). Factor loadings ranged from 0.41 to 0.92, implying that the items accurately measured the four factors of the motives subscales. The subscales demonstrated acceptable to good internal consistency in our sample (Cronbach's alpha ranged from 0.64 to 0.89). These estimates are in line with estimates of internal consistency of the motives subscales in samples of individuals with an average intelligence. In general, a Cronbach's alpha of ≥0.70 is recommended, but for scales containing less than 10 items, a Cronbach's alpha of ≥0.60 is an acceptable indicator of internal consistency (Krank et al., 2011; Loewenthal, 1996).

**Table 2:** Standardized factor loadings of the four‐factor structure of the motives items and internal consistencies

In the last 12 months, how often did you drink….	Alcohol use (*n* = 163)	Drug use (*n* = 90)
Social motives		
To celebrate a special occasion?	0.41	0.67
Because it makes social gatherings more fun?	0.70	0.82
Because it helps you enjoy a party?	0.72	0.68
Internal consistency (Cronbach's alpha)	0.63	0.76
Conformity motives		
So you won't feel left out?	0.72	0.87
To be liked?	0.63	0.83
To fit in with a group you like?	0.79	0.81
Internal consistency (Cronbach's alpha)	0.74	0.87
Coping motives		
To forget about your problems?	0.84	0.86
Because it helps you when you feel depressed or nervous?	0.89	0.92
To cheer up when you're in a bad mood?	0.75	0.82
Internal consistency (Cronbach's alpha)	0.86	0.89
Enhancement motives		
Because it's fun?	0.69	0.88
Because it's exciting?	0.67	0.78
Because you like the feeling?	0.81	0.89
Internal consistency (Cronbach's alpha)	0.76	0.88

Note

All factor loadings are significant at *p* < 0.001.

Table 3 shows that coping and enhancement motives are strongly positively correlated (*r *= 0.69 for alcohol and *r* = 0.81 for drug use). All other motives are positively related to the frequency of alcohol use and drug use and AUDIT and DUDIT scores.

**Table 3:** Correlations between the four motives and severity of substance use

	1.	2.	3.	4.	5.	6.
1. Social	–	0.52**	0.45**	0.62**	0.36**,^a^/0.40**,^b^	0.41**
2. Conformity	0.21**	–	0.53**	0.58**	0.08^a^/0.26*,^b^	0.47**
3. Coping	0.31**	0.43**	–	0.81**	0.31**,^a^/0.37**,^b^	0.63**
4. Enhancement	0.51**	0.44**	0.69**	–	0.33**,^a^/0.37**,^b^	0.59**
5. Frequency alcohol/drug	0.33**	0.23**	0.23**	0.26**	–	0.62**,^a^/0.74**,^b^
6. AUDIT/DUDIT	0.25**	0.37**	0.50**	0.49**	0.54**	–

Notes

Under the diagonal correlations for alcohol use are shown (*n* = 163), above the diagonal correlations for drug use are shown (*n* = 90).

a Correlation for cannabis use.

b Correlation for hard drug use.

* *p* < 0.05.

** *p* < 0.01.

In a third step, we examined the relationships between motives and substance use with a multivariate regression model. Results show that participants with a higher score on social motives have a higher frequency of use of substances. Relationships between the other motives and frequency of alcohol use were not significant (Table 4). In addition, participants who scored higher on conformity, coping and enhancement motives have a more severe degree of alcohol use than people with MID‐BIF who scored lower on these motives. Social motives were not significantly related to the severity of alcohol use.

**Table 4:** Multiple linear regression analysis for the relationship between motives and alcohol use

	Frequency alcohol use			Severity of alcohol use		
	*B*	*SE B*	*β*	*B*	*SE B*	*β*
Social	0.51	0.16	0.28**	0.22	0.97	0.02
Conformity	0.33	0.21	0.13	2.58	1.26	0.15*
Coping	0.15	0.17	0.09	2.91	0.99	0.27**
Enhancement	−0.01	0.21	−0.01	2.82	1.28	0.23*

Notes

Frequency of alcohol use: *R* ^2^ = 0.12; severity of alcohol use: *R* ^2^ = 0.31.

* *p* < 0.05.

** *p* < 0.01.

Table 5 shows the results for the relationship between motives for drug use and frequency of cannabis and hard drug use and the severity of drug use. Results show a positive relationship between the social motives and the frequency of cannabis and hard drug use. Participants, who have a higher score on the social motives, use more frequently cannabis and hard drug in comparison with individuals with MID‐BIF that have a lower score on the social motives. In addition, we found a negative relationship between conformity motives and frequency of cannabis use, indicating that participants with lower scores on conformity motives use cannabis more often than participants with higher scores. No significant relationships were found between other motives and frequency of cannabis and hard drug use. For severity of drug use, we found that coping motives have a significant relationship to severity of use. People with MID‐BIF who score higher on coping motives exhibit a more serious degree of use than people with MID‐BIF who score lower on coping motives. The relationship between the other motives and severity of drug use was not significant.

**Table 5:** Multiple linear regression analysis for the relationship between motives and drug use

	Frequency cannabis			Frequency hard drug			Severity drug use		
	*B*	*SE B*	*β*	*B*	*SE B*	*β*	*B*	*SE B*	*β*
Social	0.86	0.32	0.35**	0.57	0.25	0.29*	1.24	1.65	0.08
Conformity	−0.77	0.34	−0.28*	−0.07	0.27	−0.03	2.29	1.73	0.14
Coping	0.49	0.33	0.25	0.37	0.26	0.24	5.22	1.69	0.44**
Enhancement	0.16	0.40	0.08	0.03	0.31	0.02	1.29	2.01	0.10

Notes

Frequency of cannabis use: *R* ^2^ = 0.21; frequency of hard drug use: *R* ^2^ = 0.20, severity of drug use: *R* ^2^ = 0.43.

* *p* < 0.05.

** *p* < 0.01.

## 4 DISCUSSION

The aim of this study was to examine the relationship between substance use in individuals with MID‐BIF and their motives for substance use. Regarding alcohol use, this study showed that only social motives were positively related to the frequency of alcohol consumption in people with MID‐BIF. Other motives were not significantly related to frequency of alcohol use. These findings are in line with studies that show that social motives are related to non‐problematic use of alcohol (Cooper, 1994; Mezquita et al., 2010). This finding is also in line with the fact that light alcohol use is socially accepted and goes hand in hand with healthy social relationships.

In addition, we found that coping and enhancement motives were positively related to the severity of alcohol use. This is in line with studies showing that individuals who use coping and enhancement motives to drink are more likely to engage in risky and problematic alcohol use (Kuntsche et al., ). Conformity motives were also positively associated with severity of alcohol consumption for people with MID‐BIF. Previous studies showed mixed findings when it comes to the conformity motives. A possible explanation for finding an effect for conformity motives in this sample is that people with MID‐BIF want to belong to groups where the standard is to use high levels of alcohol. In these groups, there may be more social pressure to use large amounts of alcohol, while the person's own intention is to not do that (Mezquita et al., 2010).

In individuals with MID‐BIF, motives for drug use have not yet been studied in the same way and with a similar scale as motives for alcohol use. In line with the findings for frequency of alcohol use, this study showed that social motives are positively related to the frequency of cannabis and hard drug use in people with a MID‐BIF. In contrast to the findings for the frequency of alcohol use, conformity motives were negatively related to frequency of cannabis use. People with MID‐BIF who wish to conform to group norms are less likely to use cannabis. Regarding severity of drug use, coping motives were related to more severe degrees of drug use. Unlike our findings for severity of alcohol use, conformity and enhancement motives were not related to severity of drug use.

The results of this study need to be interpreted in the light of a number of limitations. First, we only included people with MID‐BIF and behaviour problems and all respondents received care from a residential facility. As a result, this sample is not representative for the population of people with MID‐BIF. We recommend to test the relationship between substance use and motives for substance use in a sample of people with MID‐BIF who do not have severe behavioural and psychiatric problems. Second, the current study only used self‐report, which may cause inadequate report of substance use (mostly under‐report or, to a lesser extent, over‐report) (see VanDerNagel et al., 2017). People with MID‐BIF have a strong tendency to agree with the proposed answers (so‐called acquiescence), especially when it comes to taboo topics, such as alcohol or drug use (VanDerNagel et al., 2017). This tendency is strengthened when questions are asked directly, which is the case in an interview or when completing a questionnaire. Most questionnaires (such as the AUDIT/DUDIT) require a certain level of knowledge of substances and a conceptual level of understanding. By developing the SumID‐Q, these limitations were taken into account. The reasonably good agreements between self‐report with the SumID‐Q and biomarker data show that the SumID‐Q generates valid data to measure substance use in people with MID‐BIF (see VanDerNagel et al., 2017). Results of the AUDIT and DUDIT show that almost half of the participants showed problematic substance use. However, cut‐off points of both instruments are based on standards for people without MID‐BIF (Babor et al., 2001; Berman et al., 2003), and despite the fact that the AUDIT and DUDIT have shown to be applicable to people with MID‐BIF (van Duijvenbode et al., 2016), “cut‐off points” for problematic use may be lower for people with MID‐BIF.

The results of this study stress the importance for including motives into personalized interventions for people with MID‐BIF and substance use instead of using an “one‐size fits all” approach. The intervention “Take it personal!” (Schijven, VanDerNagel, Lammers, & Poelen, 2014) is an example of a personalized intervention which includes personality traits and motives for substance use to connect better to the individual needs of the person. It is necessary to test whether a personalized intervention such as “Take it personal!” is more effective than an intervention which is not based on the person's personality traits and motives for substance use.

In sum, it can be concluded that both coping and enhancement motives pose a risk for problematic use of alcohol in people with MID‐BIF. For problematic drug use, coping motives are most dominant. This insight can contribute to prevention efforts or reduction of problematic substance use in people with MID‐BIF and has implications for both policies in clinical practice and for interventions for prevention and treatment in substance use. Our results show that many people with MID‐BIF in residential care are at risk for problematic substance use. Therefore, care facilities are advised to develop strict and transparent policies focusing on substance use. Moreover, it is highly important that training of staff is facilitated as well as systematic screening, diagnostics, prevention and treatment with the focus on substance use (van Duijvenbode et al., 2015). Staff members and clinicians are advised to adapt their approach to the motives for substance use. For instance, a person with the social conformity motives can be helped to become stronger in making own decisions. Instruments such as the SumID‐Q or DMQ‐R‐SF can be supportive for screening and diagnostics. Screening of motives for substance use provides a starting point for personalized and effective prevention and treatment. A person with coping motives, for instance, may benefit most optimally from an intervention that is based on cognitive‐behavioural strategies aimed at learning how to cope effectively with anxiety or negative thoughts, whereas an individual who scores high on conformity motives may benefit most likely from a social skills training (Mezquita et al., 2010). With a personalized approach of prevention and treatment, the (potentially) harmful effects of substance use disorders can be prevented.

## CONFLICT OF INTEREST

The authors declare that they have no competing interests.
